# Proteomic landscape of Alzheimer’s disease: emerging technologies, advances and insights (2021 – 2025)

**DOI:** 10.1186/s13024-025-00874-5

**Published:** 2025-07-14

**Authors:** Jay M. Yarbro, Him K. Shrestha, Zhen Wang, Xue Zhang, Masihuz Zaman, Mengqi Chu, Xusheng Wang, Gang Yu, Junmin Peng

**Affiliations:** 1 Department of Structural Biology, St. Jude Children’s Research Hospital, Memphis, TN 38105 USA; 2 Department of Developmental Neurobiology, St. Jude Children’s Research Hospital, Memphis, TN 38105 USA; 3 Department of Genetics, Genomics and Informatics, University of Tennessee Health Science Center, Memphis, TN 38103 USA; 4 Department of Neurology, University of Tennessee Health Science Center, Memphis, TN 38103 USA; 5 Department of Neuroscience, Peter O’Donnell Jr. Brain Institute, University of Texas Southwestern Medical Center, Dallas, TX 75390 USA

**Keywords:** Alzheimer’s disease, Neurodegenerative disease, Pathogenesis, Biomarker, Proteomics, Proteome, Mass spectrometry, Multi-omics

## Abstract

The advancements of proteomics technologies are shaping Alzheimer's disease (AD) research, revealing new molecular insights and improving biomarker discovery. Here, we summarize major AD proteomics studies since our 2021 review, focusing on disease mechanisms and biomarker identification. Enhanced sensitivity and throughput in proteome profiling have been driven by mass spectrometry (MS)-based approaches and affinity-based platforms (e.g., Olink and SomaScan). Emerging techniques, including single-cell, spatial, and single-molecule proteomics, provide unprecedented resolution in studying cellular heterogeneity and pathological microenvironments (e.g., amyloidome). Multi-cohort analyses of AD brain tissues have revealed consensus protein alterations (*n* = 866), identifying novel disease-associated proteins validated in functional studies (e.g., MDK/PTN, NTN1, SMOC1, GPNMB, NPTX2, NRN1, VGF, and U1 snRNP). Proteomic studies of AD biofluids have identified distinct disease subtypes, offering candidate proteins for early detection. Comparisons between human tissues and AD mouse models highlight shared pathways in amyloid pathology while underscoring limitations in recapitulating human disease. Combining proteomics with genomics enables protein quantitative trait locus (pQTL) analysis in AD, linking genetic risk factors to protein expression changes. Discrepancies between proteome and transcriptome suggest altered protein turnover in AD. Overall, AD proteomics continues to provide mechanistic insights into disease progression and potential biomarkers for precision medicine.

**Supplementary Information:**

The online version contains supplementary material available at 10.1186/s13024-025-00874-5.

## Introduction

Alzheimer’s disease is a complex and devastating neurodegenerative disorder marked by progressive cognitive decline, memory loss, and neuropathological changes [1]. It is pathologically defined by the accumulation of extracellular amyloid-beta (Aβ) plaques and intracellular neurofibrillary tangles of hyperphosphorylated tau. Recently, the U.S. Food and Drug Administration (FDA) approved several Aβ-targeting treatments, including aducanumab [2], lecanemab [3], and donanemab [4]. However, their efficacy is still limited, and their broader impact on disease progression remains uncertain [5–7], highlighting the gap in our understanding of AD pathophysiology beyond amyloid clearance.

Decades of evidence have converged on the amyloid cascade hypothesis [8], which positions Aβ as the primary driver of AD. Recent discussions in the field suggest that Aβ removal may be sufficient for drug approval and potential clinical benefit [9]. However, it is still not clear how Aβ pathology leads to downstream tau dysfunction and cell death [10]. Additionally, many patients exhibit mixed pathologies, such as cerebrovascular alteration, α-synuclein inclusion, and/or TDP-43 proteinopathy, underscoring that AD is rarely a purely “amyloid-centric” disease [11–13]. The interplay between Aβ, tau, and other comorbid processes remains a critical area of investigation.

**Fig. 1:**
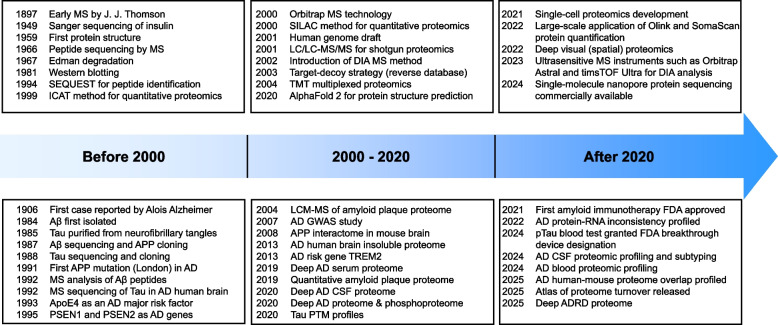
Evolution of proteomics methodologies in AD research. This schematic timeline highlights major technological advances in mass spectrometry and proteomics, categorized into pre-2000, 2000–2020, and post-2020, alongside key AD research events, particularly those related to proteomics. In 1897, J.J. Thomson pioneered early MS by demonstrating that cathode rays were composed of particles (later identified as electrons) and developing techniques to measure the mass-to-charge ratio of ions, laying the foundation for modern MS. The progression of proteomics approaches, including MS-based data-dependent acquisition (DDA), data-independent acquisition (DIA), parallel reaction monitoring (PRM), and affinity-based platforms such as Olink and SomaScan, has enabled deeper and higher-throughput proteome profiling. The transition from the DDA method, such as tandem-mass-tag (TMT), to the DIA method has improved sensitivity and throughput. Meanwhile, proteomic analysis of AD samples has evolved from single-protein studies to proteome-wide investigations across large patient cohorts

Advanced proteomics is a powerful tool for studying AD pathogenesis (Fig. 1) [14–16], and we previously published a comprehensive review on this topic in 2021 [16]. PubMed analysis shows a large rise in “Alzheimer’s disease proteomics” studies, with 3,788 articles as of March 2025, nearly double the 2,158 reported before 2021. These new studies leverage cutting-edge MS and improved bioinformatics to deepen insights into proteome dynamics in the brain tissue, cerebrospinal fluid (CSF) and blood of human patients [17–23] and animal models [24, 25]. Beyond AD, these proteomic approaches extend to other AD-related dementias (ADRD) [26], reinforcing their potential to unravel overlapping and divergent pathogenic pathways across the broader spectrum of dementias.

In this review, we present a multi-faceted survey of AD proteomics since 2021, highlighting technological breakthroughs, insights into disease pathology, functional validation of proteome-wide pathways, biomarker discovery, and multi-omics integration. We also examine comparative proteomic findings in human cohorts and mouse models to bridge translational gaps and clarify shared or distinct proteomic patterns. Ultimately, the studies can illuminate the proteomic landscape beyond Aβ and tau, the impact of comorbid pathologies, and the potential of biomarkers to diagnose, predict disease progression, and guide therapeutic response.

## Innovations in proteomics technologies

**Fig. 2:**
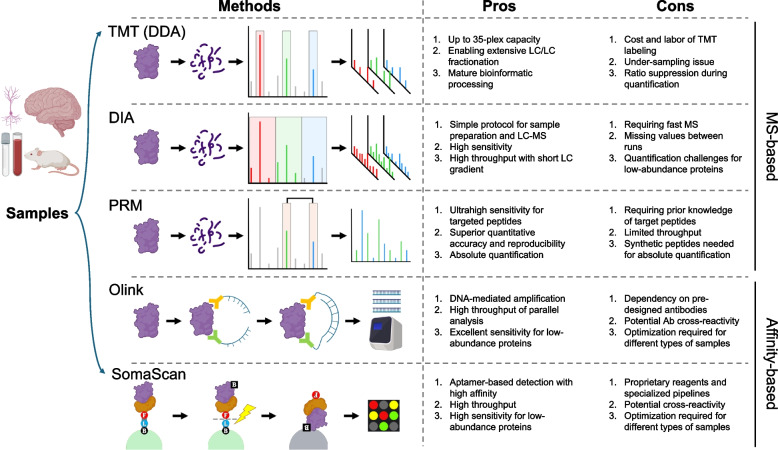
MS-based and affinity-based proteomics approaches for AD. Comparative overview of proteomic strategies employed in AD research, categorized into MS-based and affinity-based methods. MS-based approaches (e.g., DDA and DIA) provide unbiased analysis with deep proteome coverage, while affinity-based platforms (e.g., Olink and SomaScan) facilitate high-throughput detection of targeted proteins using antibodies or aptamers. Each method's advantages, such as sensitivity, throughput and multiplexing capacity, are contrasted with limitations, including missing values, quantification issues, prior target knowledge requirements, and potential cross-reactivity. Created in BioRender. Yarbro, J. [24] https://BioRender.com/ya7seph

Since its inception by J.J. Thomson, mass spectrometry has undergone transformative advancements in ionization of biomolecules and detection of the resulting ions, which significantly enhance its analytical capabilities for complex biological systems. The previous review covered key milestones in MS-based protein analysis up to 2021 [16], some of which are highlighted here (Fig. 1). In recent years, proteomics has witnessed continuous improvements, driven by innovations that boost sensitivity (detecting low-abundance proteins), throughput (speed and scale of data acquisition), and robustness (reliability of instruments and measurements). Common proteomic methods (Fig. 2) encompass isobaric labeling-based tandem mass tag (TMT) MS [27], label-free data-independent acquisition (DIA) MS, targeted parallel reaction monitoring (PRM) MS [28, 29], and affinity reagent-based high throughput methods such as Olink [30] and SomaScan [31].

The TMT MS [27] enables the quantification of over 10,000 proteins across up to 35 samples per batch when combined with peptide fractionation via two-dimensional liquid chromatography (LC), addressing the broad dynamic range of mammalian proteome [32–38]. With upgraded instrumentation, the sample requirement for TMT analysis has decreased to as low as 1 µg or less [36]. TMT proteomics is widely applied due to its high proteome coverage, extensive multiplexing, and minimal missing data [39–41]. In parallel, DIA MS has rapidly evolved, using faster Orbitrap and time-of-flight (TOF) instruments capable of identifying thousands of proteins in a single LC–MS run [42]. Ion mobility technologies, such as trapped ion mobility spectrometry (TIMS) [43–45] and high-field asymmetric waveform ion mobility spectrometry (FAIMS) [46, 47] further enhance separation power. The recent introduction of Asymmetric Track Lossless (Astral) increases proteome coverage up to 10,000 proteins with < 1 µg protein loading [42, 46–51]. Several multiplexing strategies have also been implemented for DIA analysis to enhance the throughput [52–54].

While the TMT and DIA represent untargeted methods for discovery studies, targeted MS including parallel reaction monitoring (PRM) is suited for validation studies without antibodies [28, 29]. PRM provides exceptional specificity and quantitative accuracy, typically enabling precise measurement of tens to hundreds of proteins from < 1 µg of starting material [29, 55], providing a reliable means of biomarker validation [55–61]. In addition, affinity-based platforms like Olink [30] and SomaScan [31] have become popular targeted assays for biofluids, offering highly reproducible measurements of large patient cohorts in automated microwell plate settings [62–66]. Olink employs dual antibody-based probes to detect > 5,400 proteins from ~ 6 µL of sample [63, 67–70]. Similarly, SomaScan uses DNA aptamers to measure up to 11,000 proteins with the selected kits, requiring only ~ 50 µL of sample [31, 71–74]. These platforms complement traditional MS by offering affinity-based signal amplification to detect proteins of low abundance. By harnessing the synergy between the MS and affinity approaches, researchers can obtain a more comprehensive and accurate view of the proteome.

Despite the advantages of these evolving proteomic methods, their limitations should also be considered (Fig. 2). The TMT method requires expensive labeling reagents, and its quantification is often affected by ratio suppression due to coeluting peptides [39]. Although DIA is highly sensitive and capable of identifying proteins from weak ion signals, the quantification accuracy for low-abundance proteins is often variable and prone to missing values in large-scale analyses [75]. Olink and SomaScan are constrained by the use of pre-designed affinity reagents, which may exhibit cross-reactivity, and their measurements can be affected by biofluid matrix effects or sample complexity [62]. Therefore, it is important to interpret findings from proteomic datasets with caution and a clear understanding of these limitations.

**Fig. 3:**
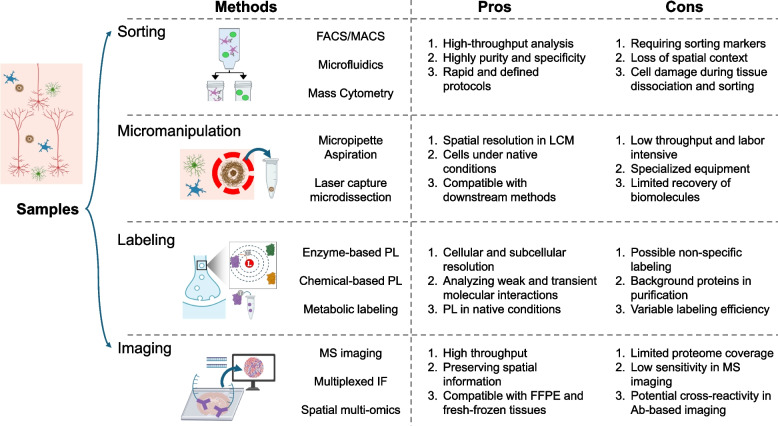
Current single-cell and spatial proteomics methodologies used in AD research. Overview of sample preparation techniques for single-cell and spatial proteomics, detailing sorting, labeling, and imaging methods. Sorting techniques such as fluorescence-activated cell sorting (FACS) and mass cytometry enable cell-type-specific proteomics, while labeling methods (e.g., enzyme-based and chemical-based proximity labeling) allow identification of molecular interactions. Imaging-based spatial proteomics techniques, including mass spectrometry imaging (MSI) and multiplexed immunofluorescence (IF), preserve spatial context and resolve cellular heterogeneity in AD pathology. Created in BioRender. Yarbro, J. [24] https://BioRender.com/rcz3b6q

Besides these bulk proteomics tools, single-cell [76, 77] and spatially resolved proteomics [78–80] promise to address single-cell heterogeneity in human disease (Fig. 3). Numerous strategies have been developed for single-cell proteomics: (i) separating individual cells by sorting, microfluidics or micromanipulation, such as micropipetting [81] or laser capture microdissection (LCM) [24, 82, 83], (ii) instead of cell isolation, labeling proteins in specific cell types or pathological area by proximity labeling (PL) [84–86] or metabolic labeling [87], followed by protein purification and MS and (iii) in situ protein imaging by mass spectrometry [88] or antibody-based fluorescence/DNA tags [89, 90]. Despite these advances, single-cell proteomics still faces challenges in cell isolation, proteome coverage, throughput, and sensitivity. Thus, few studies have utilized single-cell proteomics tools in AD. In this review, we will focus on proteomics studies from bulk AD samples and their identified targets in functional studies and biomarker validation.

## Consensus proteomic signature in AD brain tissues

**Fig. 4:**
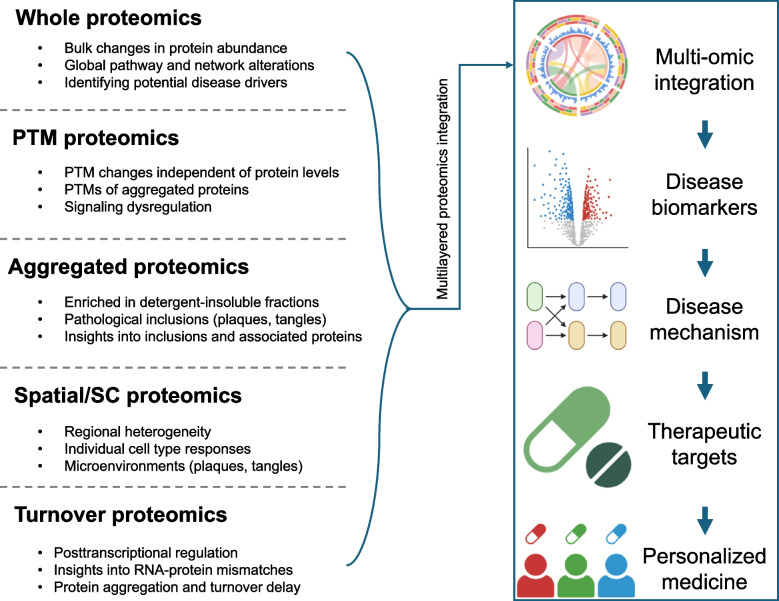
Multi-layered proteomic integration for AD pathogenesis. Illustration of the major proteomic layers investigated in AD, including whole proteome changes, post-translational modifications (PTMs), aggregated proteome, spatial/single-cell proteomics, and protein turnover. Bulk proteomic analyses provide insights into global alterations, while PTM analyses uncover dysregulated signaling events. Aggregated proteomics identifies proteins enriched in pathological inclusions (plaques, tangles), and spatial/single-cell approaches reveal cell-type-specific responses and microenvironmental interactions. Turnover proteomics enables tracking of temporal protein homeostasis disruptions, contributing to AD pathogenesis. Created in BioRender. Yarbro, J. [24] https://BioRender.com/9plux6n

The AD brain proteome has been systematically profiled at multiple levels, including the whole proteome, sub-proteomes, and protein modifications (Fig. 4). Global proteomic surveys of postmortem brain tissues reveal thousands of protein alterations in addition to hallmark features like amyloid plaques and neurofibrillary tangles [17–20, 25]. Subcellular fractionation and laser capture microdissection approaches further refine our understanding of protein signatures in subcellular and pathological regions [21, 24, 82, 91–98]. In-depth analyses of post-translational modifications continue to uncover regulatory mechanisms beyond protein levels [17, 99–102].

To date, more than 30 whole proteome datasets from AD brains have been reported and reviewed [16–18, 103]. Yarbro et al. integrated these datasets to identify 866 consensus proteins, based on the magnitude of protein changes and associated statistical significance (Supplementary Table 1) [24]. This consensus protein list was then compared with the brain proteome of commonly used AD mouse models including 5xFAD [104, 105] and APP-KI [106]. Of the 866 proteins, 654 were detected in mice, with 108 consistently altered in both models. These 108 proteins originate from diverse cell types, with microglia contributing the largest proportion (~ 40%), followed by neurons and astrocytes, along with smaller contributions from endothelial cells and oligodendrocytes. Pathway analysis revealed upregulated processes related to amyloid matrisome, cell migration, complement/coagulation, cytoskeleton organization, immune response, integrin signaling, lipid regulation, metabolism, and protein folding/proteolysis, while neurogenesis and synaptic regulation were downregulated [24]. Expanding the analysis to AD mouse models with tau or splicing pathologies (3xTg [107] and BiG [97]), approximately 42% of human AD protein changes were replicated across all four mouse models. These include well-known AD-associated proteins such as APP, APOE, TREM2, MAPT/Tau, and CLU, as well as understudied proteins like MDK, NTN1, SFRP1, OLFML3, PTPRC/CD45, SMOC1, CD180, and PTN. Comparisons between human AD tissues and mouse models reveal shared pathological pathways while also highlighting the limitations of mouse models in fully recapitulating human disease. Despite these constraints, mouse models remain important for mechanistic studies and therapeutic testing. However, selecting the appropriate model based on the molecular pathways of interest is critical for maximizing translational relevance.

Beyond whole proteome studies, recent advances in sub-proteomic approaches have provided deeper insights into AD pathology by focusing on aggregated proteome, amyloid plaques (amyloidome) and specific cellular compartments [21, 41, 91, 97, 108, 109]. Chen et al. [97] and Zaman et al. [41] analyzed Sarkosyl detergent-insoluble, aggregation-enriched fractions and identified > 8000 proteins. Using LCM-MS, Xiong et al. [91] identified > 4000 proteins in human AD plaques, while Drummond et al. [21] detected ~ 2000 plaque-associated proteins using a similar approach. More recently, Martá‑Ariza et al. [109] utilized LCM-MS to profile ~ 2000 proteins in both early onset and late onset AD plaques. To gain a comprehensive insight into key plaque-associated proteins, we performed a meta-analysis on these datasets, identifying 516 proteins consistently enriched in the amyloidome (FDR < 0.05, Supplementary Table 2). The list includes key proteins such as Aβ, APOE, MDK, HTRA1, CLU, C4A, GPNMB, COL25A1, SMOC1, NTN1, and SPON1.

In addition, sub-proteomic studies of specific cellular compartments, particularly the synaptic proteome, have shed light on the molecular mechanisms underlying cognitive decline in AD [94, 95, 110]. Recent synaptic proteomics studies revealed a progressive trajectory of synaptic dysfunction, beginning with early mitochondrial and presynaptic alterations, followed by mid-stage inhibitory synapse impairments, and culminating in the selective vulnerability of excitatory synapses in later stages [110]. Furthermore, distinct synaptic protein signatures were identified in cognitively resilient individuals compared to those experiencing dementia, highlighting pathways such as oxidative phosphorylation and serotonin signaling [94]. As sub-proteomic approaches continue to evolve, expanding their application to other critical cellular compartments, such as lysosomes and mitochondria, will further elucidate the complex molecular landscape of AD pathology.

Comprehensive proteomic profiling has uncovered critical roles for various PTMs (phosphorylation, glycosylation, etc.) in AD pathogenesis. Phosphoproteomic approaches identified numerous dysregulated kinases (e.g., MAPK [17, 24]) and hyperphosphorylated proteins implicated in neurodegeneration, notably tau, which demonstrates age-dependent phosphorylation patterns linked to PI3K-AKT-mTOR signaling disruption [111]. Additional phosphoproteomic studies in APP/PS1 models reveal that restoring phosphorylation homeostasis through chemical interventions, such as tetrahydroxy stilbene glycoside, can mitigate AD-related alterations in kinase signaling pathways [58]. However, this multifunctional natural compound may also exert its effects through kinase-independent mechanisms. Glycoproteomic analyses also identified aberrant glycosylation as a biomarker and potential driver of AD pathology [112, 113].

Tau is one of the most extensively modified proteins in neurodegeneration [114]. Using diverse MS platforms, Wesseling et al. cataloged 95 distinct PTM events (phosphorylation, acetylation, ubiquitination, and methylation) at various residues across different tau isoforms in AD [99]. These modifications appear to occur in a coordinated manner, potentially modulating tau’s biochemical properties and promoting aggregation. For example, Arakhamia et al. combined MS with cryo-electron microscopy to show that specific PTMs (e.g., ubiquitination) may influence fibril conformation and diversity [115]. Further expanding the PTM landscape, Poudel et al. identified an additional 32 PTM events (e.g., oxidation and deamidation) in AD brain tissue [116]. Moreover, similar analyses in two tauopathy mouse models (P301S and P301L) suggest that tau aggregation in mice is primarily driven by tau hyperphosphorylation, characteristic of early-stage AD, while other modifications in late-stage human AD (e.g., ubiquitination and acetylation) are absent in these models [117]. Together, these findings point to a dynamic and heterogeneous pattern of tau modification that may reflect distinct molecular states and stages of the disease.

Together, these PTM-focused investigations in AD underscore the importance of integrating diverse multi-tier proteomics approaches to capture the full complexity of molecular landscape during disease progression.

## Functional insights into proteome-wide pathways in AD

Proteomics studies have uncovered previously underappreciated changes in AD biology [16, 17, 103], prompting focused investigation of these proteins using mouse models and other systems. The proteins discussed here are selected based on multi-omics or multi-tier proteomics analyses, particularly by overlapping whole proteome and plaque proteome data, or by their potential functional significance in key AD pathways. Specifically, selection criteria include involvement in the Aβ interactome, microglia activation, synaptic deregulation, or contributions as novel components in protein aggregation.

Many matrisome proteins are enriched in amyloid plaques [24, 82, 91, 118] and some appear to play protective roles. The plaque-localized serine protease HTRA1 degrades misfolded proteins such as Aβ [119], and Chen et al. [120] demonstrated it remodels α-synuclein fibrils into non-toxic, seeding-incompetent species through a protease-independent mechanism, implicating a similar function in AD. NTN1 (and its paralog NTN3) is selectively upregulated in AD, directly binds APP [121] and Aβ [17], and protects against Aβ-induced neurotoxicity via NF-κB/Nrf2 pathways [122]. Notably, Chen et al. [123] showed that NTN1 deficiency may promote amyloid pathology and cognitive decline. SMOC1, a secreted calcium-binding protein colocalized with Aβ plaques [21, 109] and phosphorylated tau [124], was reported to interact with Aβ, delay fibril formation and alter fibril morphology, suggesting a protective role in early plaque development [124].

The paralogs MDK and PTN are consistently elevated in AD brains and directly bind Aβ, though their impacts might vary by model and disease stage [17, 18, 24, 125, 126]. For instance, Zaman et al. [126] performed comprehensive biophysical analyses demonstrating that MDK attenuates Aβ fibril assembly in vitro, consistent with earlier preliminary observations [127, 128]. Genetic deletion of MDK in the 5xFAD mouse model increased amyloid deposition and microglial activation, supporting its protective function. However, Levites et al. [125] reported that MDK or PTN overexpression in CRND8 mice increased plaque burden, suggesting complex, dosage-dependent effects. These findings collectively indicate MDK clearly impacts amyloid pathology, underscoring the need for additional mechanistic studies to reconcile the differences in different animal models.

Other Aβ-interacting matrisome proteins have distinctly pathogenic roles. Astrocyte-derived SFRP1 accumulates in AD CSF, brain, and mouse models [17, 24, 129]. It amplifies microglia-mediated neuroinflammation through sustained HIF and NF-κB activation [130] and promotes amyloidogenic APP processing by ADAM10 inhibition, increasing Aβ production and oligomerization [129]. Similarly, APOE (particularly the APOE4 allele, a major genetic AD risk factor) colocalizes with Aβ plaques, modulates Aβ aggregation and clearance in an isoform-dependent manner [131]. Kaji et al. [132] recently showed that APOE initiates amyloid pathology by seeding Aβ aggregation within the microglial endo-lysosomal compartments in a cholesterol metabolism-dependent manner. Collectively, these matrisomal proteins modulate amyloid pathology by directly interacting with Aβ and influencing microglial recruitment and activation.

Many microglia-enriched proteins identified through multi-omics analyses exhibit protective roles in AD. GPNMB is strongly upregulated in response to lysosomal stress and pathological debris [133, 134], facilitating microglial phagocytosis of Aβ and dying neurons via interaction with ATP6V1A to support lysosomal degradation and limit chronic inflammation [135]. Similarly, the DAM protein ITGAX modulates microglial phenotype and amyloid pathology, as its downregulation leads to increased Aβ accumulation and cognitive deficits [136, 137]. TREM2 is enriched near plaques and regulates microglial activation and lipid metabolism. Zhu et al. [138] demonstrated that TREM2 deletion enhances tau pathology, supporting its role in suppressing neuroinflammation and proteopathic spread. However, Dhandapani et al. [139] reported that stabilizing membrane-bound TREM2 may enhance microgliosis and amyloid deposition, indicating context-dependent effects.

Several microglial proteins have uncertain or incompletely characterized functions in AD. OLFML3 is expressed by microglia, highly upregulated in AD [17, 24], correlates positively with glymphatic clearance efficiency [140], and is associated with immune signaling and microglial activation [141], yet its precise functional role remains unclear. Similarly, PTPRC (CD45), a microglial phosphatase modulating adaptive and innate immune signaling pathways [142], is elevated in AD brain tissue and mouse models [17, 24]. CD180 is also significantly altered in AD [17, 24] and implicated in microglial immune signaling [143, 144], yet remains poorly understood.

Specific microglia-associated proteins elevated in AD have been linked to distinctly pathogenic roles. Chen et al. [145] reported the cysteine protease CTSS disrupts the blood-CSF barrier by cleaving the tight junction protein CLDN1, promoting neuroinflammation and immune infiltration. Genetic evidence from Lin et al. [146] further associates elevated CTSS with proteostasis imbalances and increased neurodegeneration risk. LSP1 is a key driver of progressive microglial inflammation from asymptomatic to symptomatic AD stages [147]. Complement components mediate pathogenic synaptic elimination via complement receptor-dependent microglial pruning of synapses [148, 149]. Collectively, these findings illustrate how dysregulated microglial proteins exacerbate neuroinflammation and synaptic loss, contributing to AD pathology.

Proteomic studies consistently demonstrate synaptic disruption as a key driver of AD pathology [17, 103]. Large-scale integrative analyses show that synaptic and vesicular pathways are among the earliest to exhibit proteomic alterations, preceding overt neuronal loss [20]. Recent studies validated specific synaptic proteins involved in maintaining synaptic integrity and function. For instance, Hurst and colleagues [150] reported that NRN1 supports cognitive resilience by stabilizing dendritic spines and protecting neurons from Aβ-induced hyperexcitability. Similarly, the downregulated neuropeptide precursor VGF [151] was shown by Beckmann et al. [152] to promote synaptic resilience, with overexpression partially rescuing memory impairment and synaptic pathology in 5xFAD mice. Zhou et al. [153] further demonstrated NPTX2 regulates complement-mediated synaptic pruning by binding C1q thereby limiting excessive microglial synapse elimination in neurodegeneration.

Beyond direct synaptic regulators, proteomic analyses have identified novel pathways affecting synaptic health, including the U1 small nuclear ribonucleoprotein (snRNP) complex. Multiple U1 snRNP components (e.g., U1-70 K, U1A) aggregate into distinct cytoplasmic tangle-like structures in AD [98]. Chen et al. [97] recently showed that a cleaved fragment of U1-70 K (N40K) dominantly disrupts U1 snRNP function, resulting in widespread splicing defects, synaptic dysregulation, and cognitive impairment in mice, thus implicating U1 snRNP dysfunction as a mechanistic contributor to AD pathology. Together, these findings highlight how proteomic approaches continue to uncover diverse, mechanistically distinct contributors to AD pathology.

## Proteomics-based biomarker discovery in biofluids

**Fig. 5:**
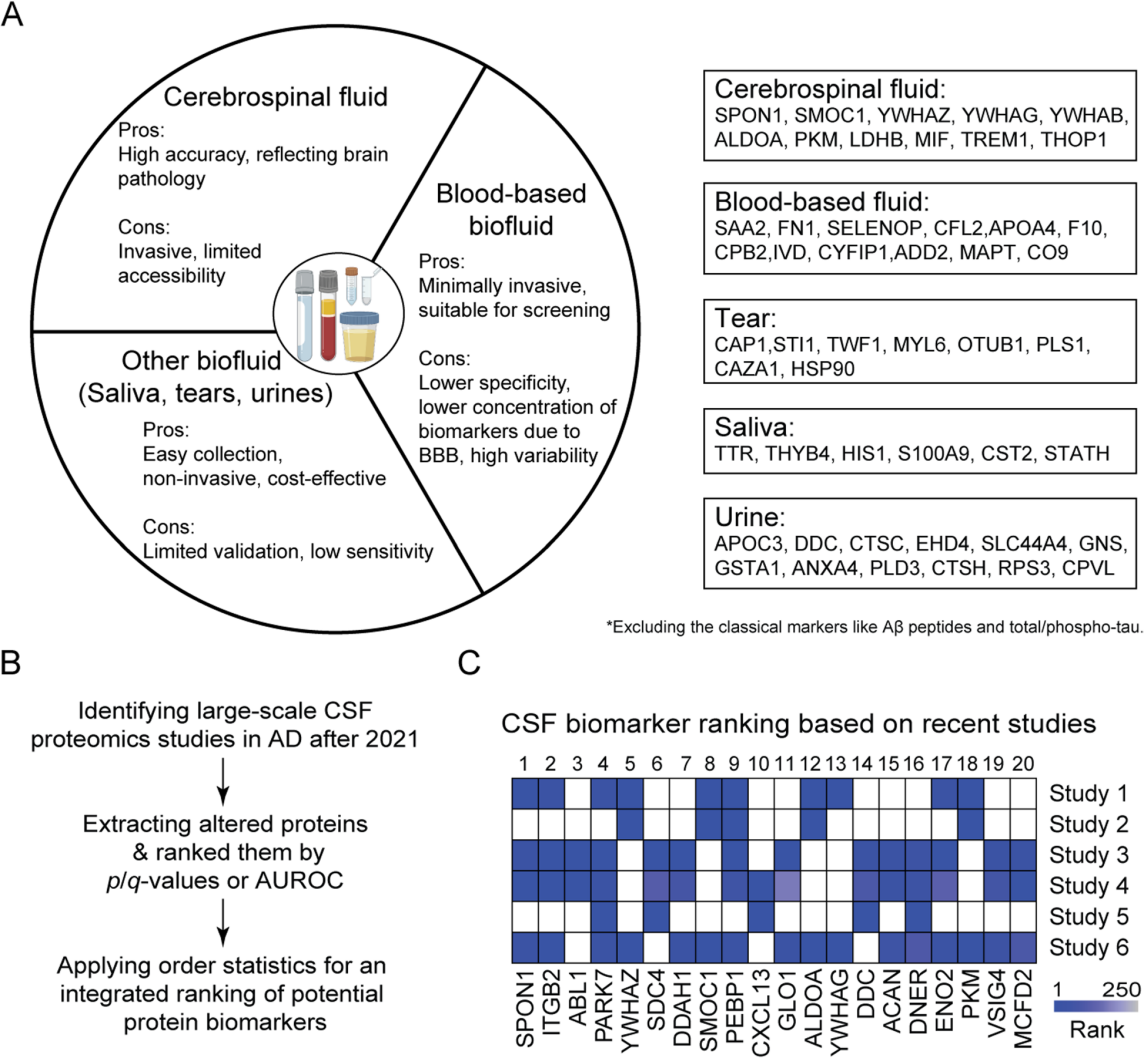
Comparative analysis of biofluid candidate biomarkers in AD. **A **Overview of CSF, blood, and other biofluid candidate biomarkers for AD detection and monitoring, highlighting their respective advantages and limitations. Key proteins identified in each biofluid category are listed, excluding classical markers such as Aβ peptides and total/phospho‐tau. **B **Workflow for the integrated ranking analysis of CSF candidate biomarkers. **C **Integrated ranking of CSF candidate biomarkers based on recent six proteomics studies in order: Johnson et al., [19]; Van Zalm et al., [154]; Del Campo et al., [155]; Del Campo et al., [156]; Del Campo et al., [157]; and Tijms et al., 2024 [158]. Created in BioRender. Yarbro, J. [24] https://BioRender.com/k2lsawa

AD pathology begins decades before clinical symptoms, underscoring the need for sensitive and specific biomarkers for early diagnosis and intervention. Biofluids such as cerebrospinal fluid (CSF), blood, saliva, tears, and urine have emerged as sources for biomarker discovery, each with inherent strengths and limitations (Fig. 5A).

The cerebrospinal fluid within the brain’s ventricular system is the most studied biofluid for AD biomarker discovery. In the 2021 review paper, meta-analysis of six deep CSF proteomics datasets identified 5,939 proteins, 476 of which showed differential abundance in AD [16]. Since then, multiple studies revealed promising biomarkers and diagnostic panels. For example, Del Campo et al. [155] analyzed CSF from 797 individuals and identified an 8-protein panel with high diagnostic accuracy. In another study, Dammer et al. [65] used TMT-MS, Olink and SomaScan to analyze CSF and plasma samples from 36 participants, identifying SMOC1, NEFL, CHI3L1, and YWHAZ as robust biomarkers. Johnson et al. [19] performed a longitudinal study of 470 individuals using PRM-MS and found that some markers such as SMOC1 and SPON1 are elevated decades before symptom onset, preceding tau and amyloid changes. Another significant study from Del Campo et al. [157] examined 534 CSF proteomes and identified a 7-protein panel that can distinguish dementia with Lewy bodies (DLB) from AD. The same group developed 12-protein panels that can differentiate between amyloid-positive and amyloid-negative individuals [156]. In a separate meta-analysis of 10 CSF datasets, Van Zalm et al. [154] pinpointed a three-biomarker panel with high predictive accuracy. Most recently, a large-scale CSF study involving 609 samples revealed five distinct AD subtypes, each linked to unique genetic and clinical profiles [158]. Another CSF proteome study reported four pseudo-trajectory protein groups during AD progression [23]. These studies introduce a new dimension of molecular heterogeneity in AD, identifying distinct subtypes that influence clinical outcomes and therapeutic responsiveness.

To prioritize biomarkers in CSF, we integrated six recent CSF datasets (Fig. 5B), including Johnson et al. [19], Van Zalm et al. [154], Del Campo et al. [155–157], and Tijms et al. [158]. Within each study, proteins were ranked by statistical significance or, when unavailable, by area under the receiver operating characteristic (AUROC) scores. We then employed order statistics across these datasets to generate an integrated ranking of candidate biomarkers, considering only proteins identified in at least two independent datasets. The integrative analysis highlighted SPON1, ITGB2, ABL1, PARK7, YWHAZ, SDC4, DDAH1, and SMOC1 as top-ranked CSF biomarker candidates (Fig. 5C).

Blood-based biomarkers offer a less invasive and more cost-effective alternative to CSF [159, 160], with notable progress in developing blood tests for Aβ and phospho-tau markers in recent years [161–165]. Beyond these classical markers, discovery-driven MS-based proteomic studies of plasma and serum identified several promising candidates [65, 166–168]. Additionally, exosomes isolated from plasma and serum further expanded the biomarker repertoire, highlighting markers such as CO9, RSU1, and ADA10 [169, 170].

Alternative biofluids such as saliva, tears, and urine offer avenues for biomarker discovery. Proteomic analyses of saliva revealed several differentially abundant proteins, including immunoglobulins, actin, metalloproteases, transthyretin, thymosin β4, α-defensins, and S100 proteins [171–173]. Tear fluid, due to its connection to neurovascular systems, was also explored for AD biomarker discovery [174]. Proteins like CAP1, STI1, TWF1, and COPS7B were reported as potential tear biomarkers [175–177]. Urine proteomics also showed potential for AD biomarker discovery [178]: a recent study identified 13-protein diagnostic panel with high diagnostic accuracy [179].

Despite these discoveries of potential biomarkers, clinical development toward routine use is a lengthy process involving multiple stages: discovery, validation, and clinical readiness [180]. In the discovery stage, candidate biomarkers are identified through high-throughput analyses in experimental or small clinical cohorts. The validation stage involves confirming the reproducibility and disease relevance of these candidates in independent and larger populations. Clinical readiness requires robust evidence of utility in real-world or clinical trial settings, often with regulatory consideration. Some of these newly discovered candidates by proteomics are undergoing validation, but none have yet reached clinical implementation. We have compiled top candidates in distinct biofluids and their clinical development status (Supplementary Table 3). The table also includes classical AD biomarkers used in clinical CSF and blood tests such as Aβ₄₂, MAPT (total tau and phosphorylated tau at sites 181, 205, 217, or 231), NFL, and GFAP, as well as relevant protein ratios (e.g., Aβ₄₂/Aβ₄₀ and pTau-217/Aβ₄₂).

Collectively, these findings hold the promise of proteomics for discovering AD biomarkers across multiple biofluids. Ongoing improvements in analytical techniques and integrative meta-analyses, coupled with validation in larger, more diverse cohorts, will help translate these discoveries into reliable clinical tests. By identifying and validating early biomarkers, researchers and clinicians can target interventions more effectively, refine patient stratification, and improve outcomes for those with AD.

## Proteomics-centered multi-omics integration in AD

Integrating proteomics with other omics data provides a comprehensive understanding of protein regulation in the AD brain. For example, protein quantitative trait locus (pQTL) analysis combines proteomics and genomics, offering critical insights into the genetic regulation of protein expression [181–185]. Since proteins are the primary effectors of cellular processes, pQTLs establish a more direct link to disease phenotypes than expression quantitative trait loci (eQTLs), which primarily influence mRNA levels [186, 187]. Moreover, comparison of proteome and transcriptome can reveal RNA-dependent and -independent protein regulation [188].

Recent pQTL studies in brain tissue have identified novel protein biomarkers associated with AD risk, disease progression, and age at onset, integrating Mendelian randomization approaches [181, 189]. Plasma-based pQTL studies have identified proteins associated with AD risk [183, 190]. Cerebrospinal fluid (CSF)-based pQTL studies provide valuable insights into protein regulation in AD [191]. For example, CSF pQTL analyses performed by Deming et al. showed that a genetic variant near MS4A6A and MS4A4A, associated with increased AD risk, was identified as a trans-pQTL that reduces soluble TREM2 levels in CSF [192]. Ferkingstad et al. demonstrated this pQTL was also correlated with lower TREM2 levels in plasma [182]. Furthermore, multi-tissue pQTL analyses have further enhanced our understanding of protein regulation across different biological compartments. A comprehensive study integrating pQTL data from brain, CSF, and plasma with genome-wide association studies (GWAS) by Hu et al. identified 30 AD risk genes, highlighting pathways enriched in immune response and glial cell proliferation [193].

In addition, protein levels are moderately correlated with RNA levels in brain tissue [188]. RNA–protein abundance discrepancies suggest significant post-transcriptional and translational regulation in AD [17]. Bai et al. uncovered a subset of Aβ-correlated proteins such as MDK and NTN1 that diverged markedly from transcriptomic predictions [17]. Johnson et al. also identified robust disease-related protein changes that showed minimal corresponding alterations at the transcript level [18]. Complementing these findings, Yarbro et al. reported that about one-third of protein changes observed in human AD brains and 5xFAD mice are independent of RNA levels. Subsequent proteome-wide turnover analysis of the 5xFAD mice [194] showed that the proteins in the amyloid microenvironment, such as Aβ-binding proteins and autophagy/lysosomal components, exhibited delayed turnover, which contributed to the protein-RNA inconsistency [24]. Moreover, others have developed multi-omic platforms that offer novel insights, such as systems pharmacology platforms for high-throughput drug repurposing in AD [195] and multi-omic resources that can be used to explore contributions from mixed pathologies in ADRD [196].

Future research will use multi-omics approaches to integrate pQTL data with transcriptomics, metabolomics, and single-cell genomics, thereby constructing causal models of AD progression [197, 198]. Spatial and single-cell proteomics will refine pQTL mapping at cellular and tissue levels. AI-driven methods will also integrate pQTL data with imaging, transcriptomics, and other omics to reveal new disease mechanisms. Identifying druggable proteins influenced by pQTLs could enable targeted therapies for AD.

## Future directions and research gaps

A deeper understanding of the proteomic landscape in AD requires new methods and a broader approach. Current studies have produced promising results on protein alterations in AD, but there are still challenges related to single-cell resolution, targeted biomarker discovery, and analysis of how protein networks change over the course of disease progression (Fig. 4). Below, we outline key areas where research should be focused.

A promising direction is combining single-cell and spatial proteomics with machine learning and AI tools [79, 199–201]. Bulk tissue studies often miss important differences between cell types [202, 203], which is particularly relevant in AD where distinct neuronal and glial populations exhibit unique pathological signatures [204]. Single-cell proteomics and AI tools can reveal this diversity and help identify rare but important cell types that drive or protect against AD pathology. AI models can also handle complex data, helping with feature selection, network analysis, and prediction [205]. These approaches can uncover new therapeutic targets for treatment.

Despite considerable advancements in fluid and imaging biomarkers, there is still a pressing need for broader, more precise biomarker panels capable of capturing disease heterogeneity. Future biomarker discovery studies should classify patients into relevant subgroups, reflecting variations in protein expression, post-translational modifications, and aggregation states. Tracking biomarkers from early to late stages will enable better monitoring of disease progression and treatment response.

Finally, addressing protein dysregulation in AD requires looking beyond individual proteins to the network level [206]. New mass spectrometric techniques and multiplex imaging platforms are improving our ability to monitor dynamic protein interactions and signaling pathways [207]. Equally important are computational modeling tools that use longitudinal data to build comprehensive network maps. These approaches capture how protein connections shift over time when mechanisms adapt or break down. Going forward, combining network-based proteomics with clinical data will be key to designing targeted treatments that disrupt pathogenic protein interactions without affecting normal cell function.

## Conclusion

Complementary to genetics and genomics, proteomic studies of Alzheimer’s disease reveal key pathological processes beyond DNA/RNA-based approaches. Large-scale proteomic analyses in human cohorts and mouse models have identified differentially expressed proteins, deepening our understanding of disease progression. Technological innovations, including mass spectrometry, machine learning analytics, and spatial profiling, have significantly improved the depth and accuracy of proteomic investigations. Multi-omics approaches highlight the molecular complexity of AD, showing that human proteomic alterations are only partially mirrored in mouse models. Future efforts will focus on refining single-cell and spatial proteomic techniques, enhancing biomarker panels for early diagnosis, and leveraging computational tools to identify truly pathogenic protein networks. These advancements will accelerate the development of novel therapeutic targets and improve patient outcomes.

## Supplementary Information

Supplementary Material 1: Supplementary Table 1. Consensus 866 differential abundance proteins (DAPs) in AD and their changes in mouse models. Supplementary Table 2. Meta-analysis of seven amylodiome datasets to identify plaque-enriched proteins in human AD brains. Supplementary Table 3. Top candidate biomarkers for AD in different biofluids.

## Data Availability

No datasets were generated or analysed during the current study.

## Abbreviations

AD: Alzheimer’s disease
CSF: Cerebrospinal fluid
MS: Mass spectrometry
LC–MS/MS: Liquid chromatography and tandem mass spectrometry
TMT: Tandem mass tag
